# Open Cyst Drainage for Reliable Decompression in a Critically Ill Neonate with Congenital Pulmonary Airway Malformation

**DOI:** 10.70352/scrj.cr.26-0246

**Published:** 2026-06-16

**Authors:** Kazuyoshi Okumura, Soma Sakamura, Insu Kawahara, Ryo Takahashi, Issei Kawakita, Momoko Ara, Shohei Honda, Akinobu Taketomi

**Affiliations:** 1 Department of Gastroenterological Surgery 1, Hokkaido University Graduate School of Medicine, Sapporo, Hokkaido, Japan; 2 Department of Pediatric Surgery, Showa Medical University Koto Toyosu Hospital, Tokyo, Japan

**Keywords:** congenital pulmonary airway malformation, emergency cyst drainage, thoracoamniotic shunting, open thoracotomy, decompression of cystic lesion

## Abstract

**INTRODUCTION:**

Congenital pulmonary airway malformation (CPAM) is a rare congenital lung lesion that is often diagnosed prenatally. Large macrocystic lesions can cause severe respiratory compromise after birth, requiring urgent decompression. Although staged management with cyst drainage followed by delayed resection has been reported, the optimal method for achieving reliable decompression in critically unstable neonates remains unclear. We report a case in which emergency open cyst drainage under direct visualization enabled secure catheter placement and reliable decompression before definitive surgery.

**CASE PRESENTATION:**

A female neonate with a large macrocystic CPAM underwent thoracoamniotic shunting during the fetal period. After birth, she developed severe respiratory failure caused by rapid enlargement of the cystic lesion and mediastinal shift. Because the precise extent of the lesion remained unclear and immediate definitive resection was considered unsafe, emergency open cyst drainage was performed. Under direct visualization, the previously placed shunt tubes were removed, and drainage catheters were securely inserted into the cystic lesions and fixed to the cyst walls. Continuous drainage achieved reliable decompression, relieved compression of the normal lung, and improved respiratory status. After sufficient lung expansion and a more accurate assessment of the lesion extent, delayed lower lobectomy was safely performed. The postoperative course was favorable.

**CONCLUSIONS:**

Emergency open cyst drainage under direct visualization may provide reliable decompression in critically ill neonates with severe CPAM. This technique may be a useful alternative to percutaneous drainage when secure catheter placement and predictable decompression are required. Effective decompression may also facilitate staged management and allow definitive resection under safer conditions.

## Abbreviations

CPAM
: congenital pulmonary airway malformation
CVR
: congenital pulmonary airway malformation volume ratio
TAS
: thoracoamniotic shunting
US
: ultrasound

## INTRODUCTION

CPAM is a rare developmental lung lesion that is usually detected prenatally.^1)^ Although most patients remain asymptomatic, large macrocystic lesions can cause severe respiratory compromise after birth because of compression of the normal lung. In neonates with acute respiratory deterioration, rapid decompression of the cystic lesion is essential to stabilize ventilation. Percutaneous cyst drainage has been used as an initial management strategy; however, in critically unstable patients, incomplete drainage or catheter malposition may result in insufficient or unstable decompression, particularly in cases following fetal intervention such as TAS. Open cyst drainage under direct visualization may offer technical advantages in such situations by enabling controlled decompression and secure catheter placement. We report a critically ill neonate with a large macrocystic CPAM who underwent emergency open cyst drainage followed by delayed lobectomy. This case highlights the usefulness of open cyst drainage for achieving reliable decompression and facilitating subsequent definitive surgery under safer conditions.

## CASE PRESENTATION

A prenatal US performed at 17 weeks of gestation revealed a right macrocystic CPAM in a female fetus. The fetus was subsequently monitored for several weeks, with no evidence of fetal hydrops or cardiocirculatory failure. However, the CPAM volume ratio (CVR) increased to 2.0. A maternal MRI scan at 20 weeks demonstrated a large cystic lesion occupying the right fetal thoracic cavity, with leftward mediastinal deviation caused by mass effect (**Fig. 1A**).

**Fig. 1:**
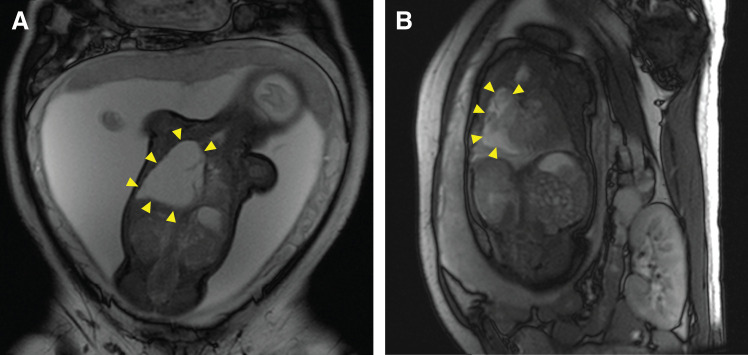
Prenatal MRI. (**A**) Maternal MRI at 20 weeks and 4 days of gestation shows a macrocystic lesion occupying the right thoracic cavity of the fetus (yellow arrowheads), with significant mediastinal displacement to the left and a CVR of 2.0. A right lung macrocystic CPAM was suspected. (**B**) Maternal MRI at 36 weeks and 2 days of gestation after TAS. The cystic lesion appears less tense, and the CVR has decreased to 1.06 (yellow arrowheads). CVR, congenital pulmonary airway malformation volume ratio; CPAM, congenital pulmonary airway malformation; TAS, thoracoamniotic shunting

At 22 weeks of gestation, the cystic lesion was punctured and 30 mL of fluid was aspirated; however, the CVR increased again. By 30 weeks, the ratio had risen to 2.46, and a decrease in the fetal heart rate was noted. At 31 weeks, US-guided TAS was attempted. Four tubes were inserted, one of which functioned as an amniotic–thoracic shunt. The remaining 3 catheters were misplaced in either the amniotic or thoracic cavity and did not function as shunts. Following tube placement, CVR gradually decreased, reaching 1.06 at 36 weeks (**Fig. 1B**).

Because of the risk of lung injury associated with TAS, as well as the potential for tension pneumothorax or rapid postnatal respiratory deterioration, delivery was planned by cesarean section at 38 weeks of gestation. A pediatric surgeon and anesthesiologist were on standby in a separate operating theater at the time of delivery.

The infant had a birth weight of 3210 g. After delivery, she developed mild cyanosis, respiratory depression, and hypercapnia. Her Apgar scores were 8 at 1 min and 9 at 5 min, and immediate postnatal surgical intervention was not required. The shunt tube inserted through the chest wall was promptly clamped, and the infant was admitted to the neonatal ICU.

No marked deterioration in respiratory status was observed. However, as anticipated prenatally, a chest radiograph revealed a right-sided tension pneumothorax with leftward mediastinal shift. The mediastinal deviation improved after thoracic drainage was performed to relieve the pneumothorax. Following chest drainage and oxygen administration, adequate ventilation was maintained.

Because the CPAM lesions identified on maternal MRI were suspected to involve multiple lung lobes, a contrast-enhanced CT scan was planned to define the exact extent of the lesion and assist with surgical planning. However, immediately before the scheduled examination, the patient developed sudden clinical deterioration. Arterial blood gas analysis demonstrated severe respiratory acidosis with marked hypercapnia, with a partial pressure of carbon dioxide exceeding 100 mmHg and a pH of 6.8. A concurrent chest radiograph showed marked enlargement of the cystic lesion with significant leftward mediastinal shift (**Fig. 2A**).

**Fig. 2:**
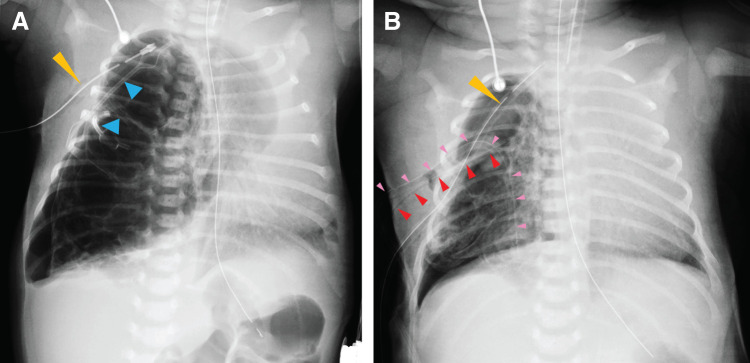
Postnatal chest radiograph. (**A**) At the onset of respiratory failure, the radiograph shows marked enlargement of the cystic lesion and severe mediastinal deviation to the left. TAS tubes (blue arrowheads) and a thoracic drain (orange arrowhead) are visible within the thoracic cavity and chest wall. (**B**) After placing cyst drainage tubes (red and pink arrowheads) into the cyst, the cyst is effectively decompressed, leading to improvement in the mediastinal deviation. TAS, thoracoamniotic shunting

As the precise anatomical extent of the lesion remained unclear and adequate gas exchange could not be maintained by the contralateral lung alone during definitive resection, initial surgical decompression was considered. If the lesion had involved multiple lung lobes, performing a single-stage lung resection during acute respiratory deterioration could have resulted in a fatal outcome because of substantial loss of surrounding normal lung parenchyma. Therefore, to prioritize rapid stabilization of respiratory status, emergency open chest cyst drainage was performed.

The incision at the previous chest drainage tube site was first extended, and the TAS tubes that had been placed during the fetal period were removed. A small incision was then made in the large cystic lesion, and an 8-Fr balloon catheter was inserted into the cyst and secured to the cyst wall with a purse-string suture using 4-0 polydioxanone. Another cystic lesion was identified on the dorsal aspect, and a catheter was placed using the same technique. Both cystic lesions appeared to be adequately decompressed. Finally, a drainage tube was inserted into the thoracic cavity. The emergency procedure was completed in 29 min, with minimal blood loss (**Fig. 2B**).

Postoperatively, continuous drainage of the cystic lesions was maintained while awaiting improvement in respiratory function and further maturation of the lungs before definitive surgery. The suction pressure for cyst drainage was generally set at −5 cmH_2_O to avoid excessive aspiration of air beyond the positive pressure provided by mechanical ventilation. As compression of the left lung gradually decreased and lung expansion improved, ventilatory support was progressively reduced, and the patient was successfully extubated on the 8th day of life.

A CT scan performed at 6 days of age demonstrated mild residual mediastinal deviation, although the cystic lesions had begun to decrease in size. During this period, air was typically aspirated from only 1 cyst, suggesting communication between the 2 cystic cavities (**Fig. 3A** and **3B**). A follow-up CT scan on postnatal day 23 showed marked reduction of the cystic lesions, improved expansion of the normal lung, and substantial resolution of mediastinal deviation (**Fig. 3C** and **3D**).

**Fig. 3:**
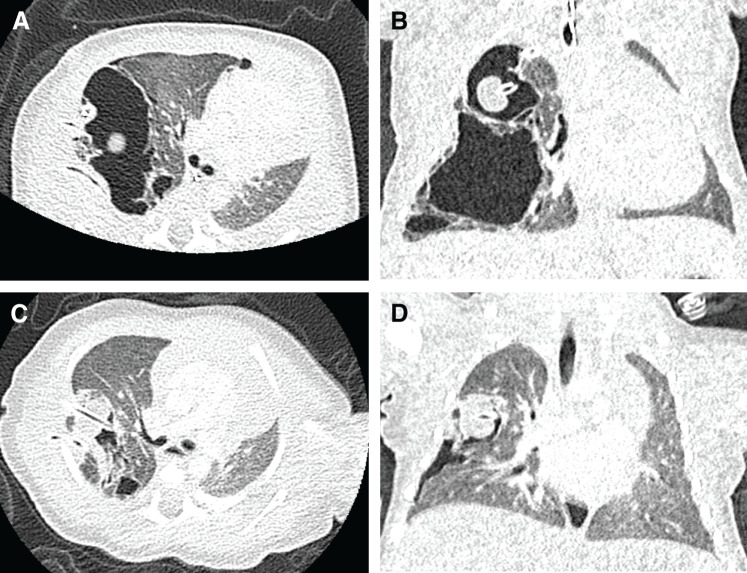
Chest CT scans after initiation of cyst drainage. (**A**, **B**) Day 4 after the initiation of cyst drainage. Both drainage tubes are positioned within the cystic lesion. (**C**, **D**) Day 21 after the initiation of cyst drainage. The cystic lesions are significantly reduced in size, with marked improvement in normal lung expansion and mediastinal position.

An open thoracotomy was selected instead of a thoracoscopic approach for several reasons. The lesion was large and suspected to involve multiple lung lobes. In addition, prior fetal and postnatal interventions increased the likelihood of intrathoracic adhesions. Definitive surgery was therefore performed on Day 36 of life after sufficient expansion of the left lung had been achieved. Intraoperatively, minor adhesions were carefully dissected. The CPAM lesions were confined to the lower lobe, with no cystic involvement of the upper or middle lobes. A lower lobectomy was performed, and the interlobar plane was created using linear staples (**Fig. 4A**).

**Fig. 4:**
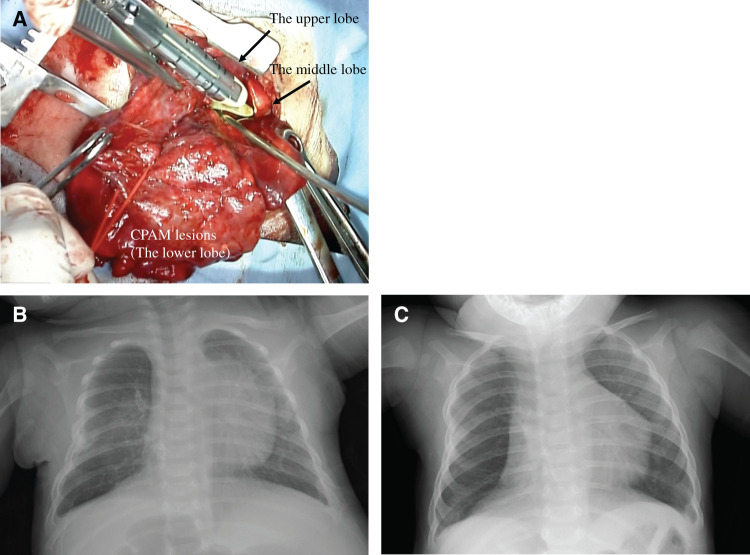
Intraoperative findings and postoperative chest radiograph. (**A**) Intraoperative findings demonstrated that the CPAM lesion was confined to the lower lobe, and the interlobar plane was divided using a linear stapler. (**B**) On POD 15, the radiograph shows excellent expansion of the left and right lungs. (**C**) A chest radiograph obtained 6 months postoperatively showed good lung expansion with no recurrence of cystic lesions. CPAM, congenital pulmonary airway malformation

The patient was extubated on the day after surgery but required reintubation shortly afterward because of worsening respiratory status. With treatment that included steroid therapy and epinephrine, she was successfully extubated 6 days after surgery. A chest radiograph obtained on POD 15 demonstrated good expansion of both lungs, and supplemental oxygen was no longer required (**Fig. 4B**). She was discharged on POD 20.

A follow-up chest radiograph performed 6 months after surgery showed satisfactory lung expansion (**Fig. 4C**). The patient remained free of respiratory complications until transfer to another hospital 8 months after surgery because of relocation.

## DISCUSSION

Large CPAM lesions can increase intrathoracic pressure and impose a significant burden on fetal and neonatal respiratory and circulatory function.^2,3)^ In symptomatic neonates presenting with respiratory compromise, prompt intervention is required to relieve compression of the normal lung and stabilize ventilation. In such situations, achieving rapid and reliable decompression is critical, as incomplete or unstable drainage may result in persistent or recurrent respiratory deterioration.

In the present case, fetal intervention was selected because the lesion was macrocystic, the CPAM volume ratio progressively increased, and fetal heart rate abnormality was observed. Although avoidance of fetal intervention with earlier delivery and immediate neonatal surgery might have been considered, surgical intervention before functional pulmonary maturation was considered high-risk. Previous reports of neonatal definitive surgery for severe CPAM, particularly thoracoscopic surgery, have generally involved patients in whom gestation was successfully prolonged to near term and respiratory status was relatively stable.^4,5)^ Therefore, prolongation of gestation following TAS was considered beneficial in the present case.

Percutaneous transthoracic cyst drainage has been described as an initial management strategy in neonates with large macrocystic lesions.^5)^ However, several technical limitations have been reported. Catheter misplacement may occur when the catheter remains outside the cyst cavity, and the elastic and mobile nature of cyst walls may prevent successful catheter advancement. In addition, accurate control of puncture angle and depth is technically demanding, and catheter misplacement may increase the risk of lung injury.^6,7)^ These limitations suggest that percutaneous drainage may not always provide reliable decompression in emergency situations requiring immediate and predictable reduction of intrathoracic pressure.

Open cyst drainage under direct visualization offers several technical advantages over percutaneous approaches. First, in patients who have undergone fetal interventions such as TAS, previously inserted catheters may remain within the thoracic cavity or become displaced.^8)^ Blind percutaneous puncture in such situations carries a risk of catheter interference or additional lung injury. In contrast, open drainage enables direct identification and management of existing catheters, allowing safe manipulation or removal while achieving controlled decompression.

Second, neonatal cyst walls are often extremely thin and fragile. Percutaneous drainage may be associated with catheter dislodgement, positional instability, or worsening air leakage.^6)^ Open drainage through a small incision allows direct handling and fixation of the cyst wall, enabling secure catheter placement and reducing the risk of recurrent collapse. This represents a particularly important advantage for maintaining continuous and stable decompression.

Third, in emergency settings, reliable and predictable decompression is essential for rapid stabilization of respiratory function. Catheter malposition or insufficient drainage may lead to further clinical deterioration.^7)^ Open drainage provides a controlled method for achieving decompression under direct visualization, which may be particularly advantageous in critically unstable neonates.

In the present case, the patient developed severe acute respiratory acidosis with marked hypercapnia and mediastinal shift, necessitating immediate intervention. To avoid unnecessarily extensive lung resection and to ensure secure catheter placement and predictable decompression under direct visualization, open cyst drainage was selected. This approach enabled effective reduction of intrathoracic pressure and rapid improvement in respiratory status.

Importantly, stabilization of respiratory function through reliable decompression allowed time for gradual re-expansion of the compressed normal lung and a more accurate assessment of lesion extent. Initial imaging suggested possible involvement of multiple lobes; however, after decompression and lung expansion, the lesion was ultimately confirmed to be confined to the lower lobe. As a result, definitive resection could be performed under more favorable conditions, and lower lobectomy alone was sufficient to achieve complete removal of the lesion.

This observation, consistent with previous reports, suggests that, in selected cases, staged management following effective decompression may facilitate safer surgical planning and help avoid unnecessarily extensive lung resection, thereby contributing to preservation of normal lung parenchyma.^9,10)^

However, open cyst drainage requires surgical exposure and generally requires more time and preparation than percutaneous drainage. Therefore, this approach may not be feasible in patients with profound physiological instability who require immediate decompression. Careful patient selection and assessment of the urgency of intervention are essential when considering open drainage in emergency settings.

## CONCLUSIONS

Emergency open cyst drainage under direct visualization may provide reliable decompression in critically ill neonates with CPAM. This approach may represent a useful alternative to percutaneous drainage when secure catheter placement and predictable decompression are required. In addition, effective decompression may facilitate subsequent staged management and allow definitive surgery under safer conditions.

## DECLARATIONS

### Availability of data and materials

The data supporting the findings of this study are available from the corresponding author upon reasonable request, in accordance with patient privacy restrictions.
